# Evaluating the More Suitable ISM Frequency Band for IoT-Based Smart Grids: A Quantitative Study of 915 MHz vs. 2400 MHz

**DOI:** 10.3390/s17010076

**Published:** 2016-12-31

**Authors:** Ruben M. Sandoval, Antonio-Javier Garcia-Sanchez, Felipe Garcia-Sanchez, Joan Garcia-Haro

**Affiliations:** Department of Information and Communication Technologies, Universidad Politécnica de Cartagena (UPCT), Campus Muralla del Mar, E-30202 Cartagena, Spain; ruben.martinez@upct.es (R.M.S.); felipe.garcia@upct.es (F.G.-S.); joang.haro@upct.es (J.G.-H.)

**Keywords:** smart grid, internet of things, wireless sensor networks, evaluation, propagation

## Abstract

IoT has begun to be employed pervasively in industrial environments and critical infrastructures thanks to its positive impact on performance and efficiency. Among these environments, the Smart Grid (SG) excels as the perfect host for this technology, mainly due to its potential to become the motor of the rest of electrically-dependent infrastructures. To make this SG-oriented IoT cost-effective, most deployments employ unlicensed ISM bands, specifically the 2400 MHz one, due to its extended communication bandwidth in comparison with lower bands. This band has been extensively used for years by Wireless Sensor Networks (WSN) and Mobile Ad-hoc Networks (MANET), from which the IoT technologically inherits. However, this work questions and evaluates the suitability of such a “default” communication band in SG environments, compared with the 915 MHz ISM band. A comprehensive quantitative comparison of these bands has been accomplished in terms of: power consumption, average network delay, and packet reception rate. To allow such a study, a dual-band propagation model specifically designed for the SG has been derived, tested, and incorporated into the well-known TOSSIM simulator. Simulation results reveal that only in the absence of other 2400 MHz interfering devices (such as WiFi or Bluetooth) or in small networks, is the 2400 MHz band the best option. In any other case, SG-oriented IoT quantitatively perform better if operating in the 915 MHz band.

## 1. Introduction

The Internet of Things (IoT) has emerged as a potential game changer for the way people interact with technology on a daily basis, seamlessly connecting the physical and digital worlds by using the Internet. Although the general public strongly associates the IoT with concepts such as “home automation” or “home appliances”, the truth is that the IoT aims at being (and has the potential to be) employed pervasively in many other environments—from industrial machinery to the water supply chain, Smart Cities (SC), Smart Grids (SG), etc. One of the main additional advantages of applying IoT technologies to these industrial fields is that we can rapidly improve infrastructure or industry performance in an affordable and cost-effective way [1].

Among these industrial fields, one particularly excels for its potential to have a particularly positive impact on the society via the application of the IoT: the SG [2]. The SG is called to revolutionize the way electricity is generated, distributed and finally consumed by every of us. By the inclusion of two-way communication systems, the electricity grid will become a more efficient, reliable, and flexible infrastructure [3], and although there are other technologies that could also be applied in the SG (like traditional centralized, wired communications), inexpensive deployment costs and the flexible and easy-to-configure nature of the IoT make it one of the most appropriate approaches for renovating the traditional power grid [1].

Nonetheless, like most Information and Communication Technologies (ICTs), the IoT strongly relies on wireless communications as its main enabling technology. Thus, deep insights into this form of transmission are of paramount importance to unleash the true potential of the IoT – and this is even more so in the SG where the complex and heterogeneous environments they operate in play a major role in the communication process. Many of these underlying wireless technologies that IoT systems employ, have been directly borrowed from Wireless Sensor Networks (WSN) or Mobile ad hoc Networks (MANET), as they originally paved the way for the emergence of these new paradigms [4]. Thus, communication technologies employed by most IoT devices are those used in WSNs or MANETs—either Mobile Telecommunications Technologies (MTT), such as 3G/4G [5,6], Cognitive Radio (CR)-based solutions [7,8,9] or technologies operating in the 2400 MHz ISM unlicensed band, such as WiFi, ZigBee or Bluetooth [10,11,12].

Since IoT-based SG should strive for high levels of self-independence and cost-effectiveness, it is not generally advisable to rely on private or licensed bands (such as those used in MTT). Unexpected and uncontrolled MTT failures or high expenditure costs can make deployments economically or technically unfeasible. On the other hand, although wideband Cognitive Radio (CR) approaches have also been thoroughly studied in the literature, the increased cost multi-band transceivers makes this approach unfeasible if intended to be used in every device of an IoT network – note that the IoT is envisaged to consists of more than hundreds or thousands of devices [13]. For these reasons, most battery-powered IoT devices that are conceived to operate in SG use ISM bands [14], generally the 2400 MHz one, and employ low-power standards such as the 802.15.4, 6LoWPAN or ZigBee [15,16,17].

Although there are other ISM unlicensed bands that have also been exploited since the emergence of the WSN (apart from the 2400 MHz), the evolution to the IoT left them practically unused. The reason to do so was the higher transmission rate that the aforementioned standards provide for the 2400 MHz band (250 Kb/s) along with lower power consumption (orders of magnitude smaller than other non-low-power 2400 MHz standards, such as WiFi, or other ISM bands such as the 5 GHz).

Another key aspect known by the research community is that most of IoT-based SGs do not make extensive use of the network; they transmit information very occasionally [18]. This is particularly true in some segments of the SG such as substations or solar plants/wind farms. There, the different elements are monitored (via IoT devices) and their information aggregated (once sent to a gateway) before being transferred to a central processing station (through wired internet networks), where data are stored and analyzed. Therefore, the work cycle of IoT devices operating in such environments, generally boils down to sensing some physical parameter (s) (temperature, pressure, humidity, etc.), interact with other devices periodically or on demand, and finally, provide some form of added-value service (such as a complete report on the status of a specific transformer or the energy generation rate of some windmills). This gives rise to a very relaxed duty cycle; allowing devices to remain silent and dormant most of the time in order to reduce their power consumption. Therefore, in some IoT networks deployed in SG, there is no pressing need to employ “high” data rates, and it seems logical to explore the advantages and disadvantages of using other unlicensed standardized ISM bands with smaller bandwidth.

Off-the-shelf IoT devices that make use of the 802.15.4/6LoWPAN/ZigBee low-power standards operate in one of the three following bands: 2400, 915 or 868 MHz [19]. While the two former bands have more than ten channels in which IoT devices may work; the 868 MHz band only offers one channel. This issue may represent an unsurmountable obstacle in scenarios where the large number of IoT devices may force the use of channel-assigning algorithms to prevent an increase of interferences and collisions [20,21,22]. Therefore, as a smaller-bandwidth alternative to the 2400 MHz band, the 915 MHz one may represent an option for IoT-based SGs worth studying. As a matter of fact, although such a band works at slower rates (40 Kb/s), it has some appreciable benefits; among them, we should note two that are crucial in the SG arena: longer coverage and less power consumption.

In this context, the main contribution of this paper is an exhaustive and quantitative analysis of the impact of the working frequency band (915 MHz or 2400 MHz) on an SG-oriented IoT network. We have focused our study on an SG’s generic-enough substation for two reasons: firstly, it is a representative scenario in which the SG may benefit from smaller transmission bandwidths. And secondly, very few works have studied this environment in depth from the wireless communication perspective [23]. Given the complex nature of such a scenario (full of large metallic structures and high levels of electromagnetic noise), we have characterized it for the two aforementioned bands. Hence, the second contribution of this work: a dual-band propagation model focused on IoTs operating in SG’s substations. This second contribution is critical as reckoning with an accurate propagation model is of paramount importance in evaluating different IoT environments with high precision.

To evaluate both bands, we have incorporated this propagation model in TOSSIM [24], a very well-known and extensively employed simulator for IoT/WSN networks. We have also extended TOSSIM with the ability to simulate the 915 MHz band and the ability to support different line-of-sight visibility conditions for IoT devices deployed in the same network. This way, and with the help of several information-extraction scripts programmed for this work, we have analyzed and derived valuable information from different simulated environments. Both, the propagation model and the extensions/tools for the TOSSIM simulator have been made publicly available in [25].

It is worth mentioning that although this paper is focused on analyzing and quantifying the performance of the 915 MHz and 2400 MHz bands, the results derived for the 915 MHz band are translatable to the 868 MHz one. This is due to the small frequency gap between these two bands (60 MHz in the worst case) and the relatively large coherence bandwidth (CBW) of the channel (in average, 94.20 MHz). The coherence bandwidth is defined as the range of frequencies over which two frequency components are likely to experience similar amplitude fading [26]. Hence, two bands with a maximum frequency gap smaller than the channel’s CBW will be strongly correlated (in terms of the propagation phenomena experienced).

The final aim of this work is thus, to empower the process of choosing the working frequency band for SG-oriented IoT networks with enough quantitative metrics (such as battery consumption or network delay) to shift it from a premade industry decision to a requirements-based decision. The rest of the paper is organized as follows. Section 2 reviews previous related work. Section 3 details the propagation model, the methodology followed and the improvements made on the TOSSIM simulator. In Section 4 we present and discuss the results obtained for different configurations of interest. Finally, Section 5 summarizes the main conclusions.

## 2. Related Work

The application of the IoT to the SG is a relatively new field, nonetheless underpinned by the wide experience of the academic community in WSN and MANET [4,16]. Parikh et al. [14] looked into different enabling technologies available for the SG, paying special attention to the benefits and drawbacks of each one, whereas [1] directly explored the architecture of the future IoT-based power grids as well as the key technologies that make this possible. Similarly, [27] analyzed the positive impact of the IoT on the energy domain, highlighting the sheer potential of such innovation in our daily lives. Finally, [28] elaborated on the positive impact that Fog Computing—a computing paradigm closely related to the IoT—may have on the Smart Grid arena. All in all, although these works study the enabling technologies in depth, they do not address the actual impact on the SG-oriented IoT of communication issues (such as the working frequency band).

Regarding the study of wireless propagation, Kusy et al. [29] studied the effects on the percentage of packet losses of incorporating two radio interfaces in WSN nodes: one working at 2400 MHz and another in the 915 MHz band. Although very accurate and illustrative, the work was focused on WSNs operating in forests, which does not share many similarities with the IoT for SGs. Hrovat et al. [30] analyzed two Smart City critical infrastructures’ propagation environments in the 400 MHz, 868 MHz and 2400 MHz bands. However, this study concentrates on elaborating empirical propagation models and does not evaluate/quantify their work with any figure of merit that may demonstrate the impact of the obtained models on actual IoT networks. Along the same lines, reference [31] proposed an accurate industrial propagation model capable of predicting low-level propagation characteristics with high accuracy in different frequency bands, whereas reference [32] provided near-ground path loss measurements for wireless deployments in indoor corridors for the different working frequencies. In general terms, the available literature—and these two studies in particular—focus on studying the low-level propagation phenomena applicable to different frequency bands, irrespective of the impact of such phenomena to the upper technologies (802.15.4, WiFi, Bluetooth, etc.) or the overall context (WSN, IoT, etc.).

To the best of our knowledge, there is no scientific literature that jointly analyzes, quantifies and compares the effects of different working frequencies on an IoT deployment. Similarly, we have not found any study that proposes a dual-band propagation model for the Smart Grid.

## 3. Methods and Tools

**Figure 1:**
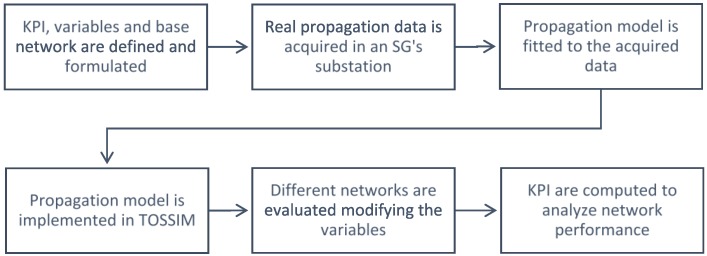
The six-step process followed to extract the propagation data, model the environment, define the network and finally, simulate and evaluate it.

In this section we elaborate on the methods and tools required to evaluate the impact of the working frequency band on an IoT-based SG. The process followed is sketched in Figure 1, which synthetizes the six steps (blocks) required to quantitatively simulate the wireless network under study with high accuracy.

### 3.1. Selected Variables and KPIs

In order to quantify the impact of the frequency band on an IoT network operating in an SG scenario, we consider three Key Performance Indicators (KPIs): the Packet Reception Rate (PRR), the Mean Network Delay (MND), and the Power Consumption per Packet Transmission (PCPT). PRR computes the percentage of issued packets that reach the receiver, illustrating how lossy the network is at that frequency. MND accounts for the mean time a given packet takes to arrive to the final receiver; if such a packet gets lost, retransmissions will be issued until it is received and accounted in the MND (the retransmission protocol employed is a simple stop-and-wait Automatic Repeat request or ARQ algorithm [33] which makes use of ACKs and timeouts to ensure packet reception). This metric reflects the average response time (or responsivity) of the network. Regarding the PCPT, only the consumption derived from the network interfaces was considered. Thereby, we separated the CPU consumption (which is technology-dependent, and thus, not relevant to this work) from the radio consumption (which is highly band-dependent). To evaluate this figure, we have considered the currents drawn (as per specified in their respective datasheets) of two devices that have been extensively employed in the literature: the CC2420 (a radio transceiver for the 2400 MHz band) and the CC1000 (working in the 915 MHz band), both making use of the low-power 802.15.4 standard.

**Figure 2:**
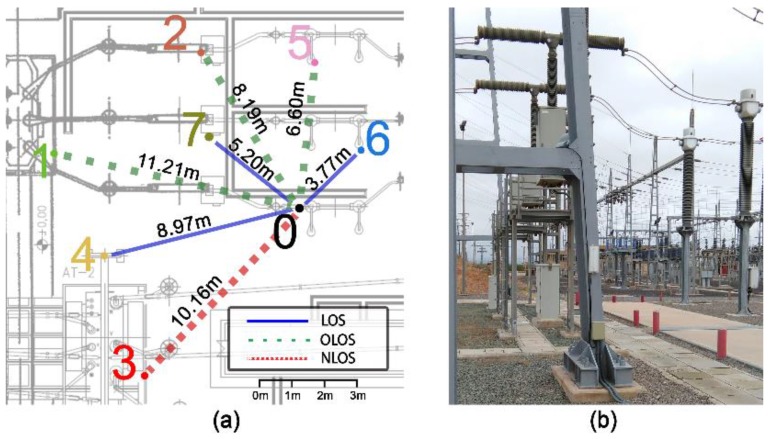
(**a**) Simulated base network (**b**) SG substation where the data was acquired.

By defining these three KPIs, we can fully evaluate the performance of the network under several different situations. To study how each band responds to such conditions, three variables have been considered in the analysis: (i) level of background noise in the working band, (ii) packet length, and (iii) mean distance between nodes (${\bar{D}}_{in}$). With regard to the noise, it is widely known that the 2400 MHz band is heavily used by many other wireless technologies apart from the 802.15.4 standard: Bluetooth, WiFi, cordless phones, etc. Thus, to study the effects of this phenomenon we have considered two scenarios: one in which the network is operating in a relatively interference-free area (hereinafter referred as Scenario 1) and one surrounded by many other 2400 MHz wireless devices (Scenario 2). Also, the packet size has been varied by modifying the payload length. By increasing it from 6 to 12, 18, and 24 bytes, we obtain a total packet length of 25, 31, 37, and 43 bytes, respectively (we considered the overhead of the IEEE 802.15.4 standard valued in 19 bytes). Finally, the mean distance between nodes has been controlled by a scaling factor α that multiplies the default distance between pairs of nodes, thus varying the mean inter-node distance (${\bar{D}}_{in}$). A scaling factor of one (α =1) corresponds to the base network (see Figure 2a) that presents a ${\bar{D}}_{in}$ of 7.73 m.

Figure 2a represents the base network on which the variations described above have been tested. Such a network consists of eight devices in charge of monitoring different aspects of an SG’s substation and of providing added-value services by exchanging information among them (e.g., notifications of an unexpected global increase in temperature). Devices are arranged under different line-of-sight conditions: line-of-sight visibility (LOS, blue line), obstructed line-of-sight visibility (OLOS, green line) and non-line-of-sight visibility (NLOS, red line). Furthermore, they are deployed following a star topology with the explicit objective of not considering the effects of multi-hop algorithms on final results. This decision makes our conclusions much more algorithm-independent. Carrier Sense Multiple Access with Collision Avoidance (CSMA/CA) interferences produced by other neighboring devices (neglected in many other works) have also been studied and accounted for.

### 3.2. Propagation Model

To be able to evaluate the behavior of the communication network presented above, we derived and implemented an accurate propagation model that was finally programmed in the TOSSIM simulator. This task was decomposed into two steps: first, we acquired real propagation data in an SG substation of Iberdrola (a Spanish energy provider; see Figure 2b) by means of a high-end Vector Network Analyzer (the E5071B – VNA, Agilent Technologies^®^, Santa Clara, CA, USA). Finally, a mathematical model that approximates the propagation behavior of the SG was chosen (see Equations (1) and (2)) and fitted to the acquired data. This two-fold approach guarantees that if the fitting process is thorough, the propagation model will replicate the original data, and thus, an SG environment.

Arguably, the most important propagation phenomenon when simulating narrow-band communications (such as the 802.15.4 standard) is the relation between the received power and distance to the transmitter. Thus, we have focused on accurately replicating this relation. To do so, we distinguish three visibility conditions between device pairs: LOS, OLOS and NLOS. Differentiating these scenarios is of paramount importance as propagation losses have been proven to be strongly influenced by the visibility conditions of communication links [17,31,34]. In order to model this phenomenon, we have first obtained a series of measures (via the VNA) under the aforementioned visibility conditions: the VNA generates a known signal that travels the wireless medium before reaching the receiver. Then, the differences between the transmitted and the received signal are analyzed and lastly fitted to a propagation model. This procedure is repeated for the following distances: 1, 2, 5, 10, 15, 20, 25, and 30 m. In order to gain statistical confidence, for each distance, we have acquired up to 30 individual measurements over an area of λ/2 (i.e. half of the wavelength, which translates to 32.8 cm and 12.5 cm for the 915 MHz and 2400 MHz respectively). This is a fairly common method in characterizing propagation environments [31,35] and is carried out with the intention of make the derived propagation model as much general as possible.

**Figure 3:**
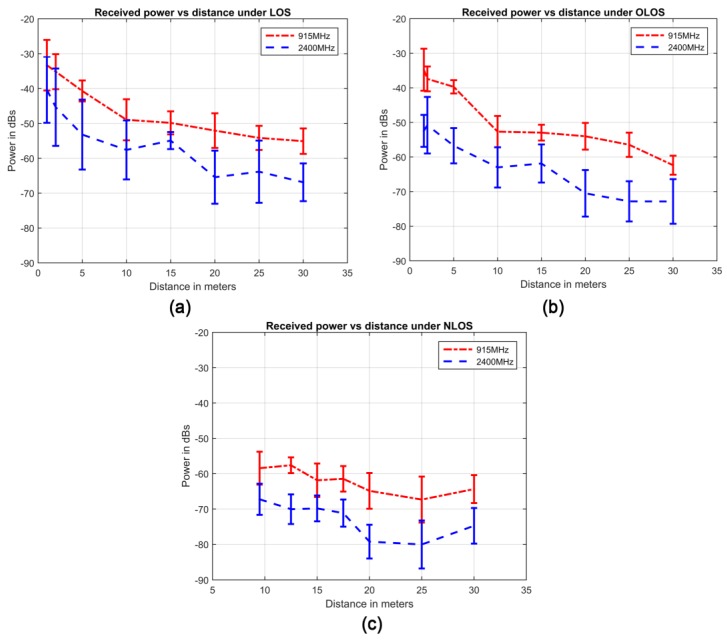
Received power vs. distance for (**a**) LOS, (**b**) OLOS, and (**c**) NLOS visibility conditions in an SG environment. 95% confidence intervals are also included.

This procedure is rerun for both bands: the 915 MHz and the 2400 MHz ones. Figure 3 above shows the received power vs distance for the different visibility conditions and frequency bands (along with the 95% confidence intervals).

As explained before, the main purpose of the propagation model is to replicate the behavior of the acquired data (presented in Figure 3) under the three different visibility conditions. The first significant phenomenon in Figure 3a–c to reproduce is the progressive deterioration of communication as the receiver moves away from the transmitter (i.e., less power is received when the distance increases ${\bar{D}}_{in}$ grows). This and other important phenomena, such as the presence of greater losses in NLOS than OLOS/LOS or larger losses in higher frequencies, can be adequately characterized by a very well-known propagation model: the log-normal shadowing path-loss model (LNSPL) [36].

The LNSPL (Equation (1)) models the dependency of received power on distance by considering the distance *d* at which we evaluate the path losses. It permits modulating the effect of this phenomenon (the rate at which the received power decays with distance) by adjusting the path loss exponent *n* for different scenarios, frequencies and visibility conditions. Furthermore, the variance over the mean received power is adjusted for each situation by a zero mean Gaussian random variable $\left({Χ}_{\sigma }\right)$ – which represents the variability of the received power over the expected value. Finally, in Figure 3 it can also be observed that the received power at the first measured distance is not zero, but it has an offset that depends on the conditions evaluated. This effect is considered in the LNSPL model by the base path losses (BPL) at a reference distance *d*_0_. These base path losses are generally calculated by the Free Space Path Loss (FPSL) formula (Equation (2)) where *d*_0_ takes the value of 1 m for convenience and comparability of results and λ is the wavelength of the working frequency [36]:

(1) $$PL{\left(d,\;n,\;\sigma \right)}_{dB}=BPL\left(d_{0}\right)+10\;n\log_{10}\left(\frac{d}{d_{0}}\right)+{Χ}_{\sigma }$$

(2) $$FSPL\left(d_{0},\lambda \right)=10\log_{10}{\left(\frac{4\;\pi \;d_{0}}{\lambda }\right)}^{2}$$

Once the appropriate propagation model is chosen, it is adjusted to replicate the acquired data (Figure 3). To do so, we have minimized the sum square error (SSE) between the real data and the propagation model (Equation (3)). Mathematically the SSE is defined as follows:

(3) $$SSE=\overset{n}{\underset{i=i}{\sum }}{\left(y_{i}-\hat{y_{i}}\right)}^{2}$$

where $y_{i}$ represents the real data and $\hat{y_{i}}$ the predicted data for a given measure *i*. In turn, $\hat{y_{i}}$ is computed by Equation (1) and $y_{i}$ is obtained by calculating the difference between transmitted and received signal’s power in the VNA. The value of Equation (3) is then minimized by Gradient Descent, a well-known iterative optimization algorithm extensively used in the IoT and WSN arena [37,38,39]. Such an algorithm is able to find (in this case) a pair of *n* and σ that reduces the differences between predicted and real data. This approach is particularly suitable for the LNSPL model as it exhibits good mathematical properties such as differentiability and convexity (features desirable to reach the global minimum of the SSE in the network under study). Table 1 shows the exact values of the LNSPL model for the three visibility conditions and the two working frequency bands as well obtained following the described procedure.

It is worth mentioning that, as expected, the path-loss exponents are greater for the NLOS than OLOS or LOS communications. On the other hand, the 915 MHz frequency band, gives rise to more predictable links (lower values of σ) in comparison with the 2400 MHz band.

**Figure 4:**
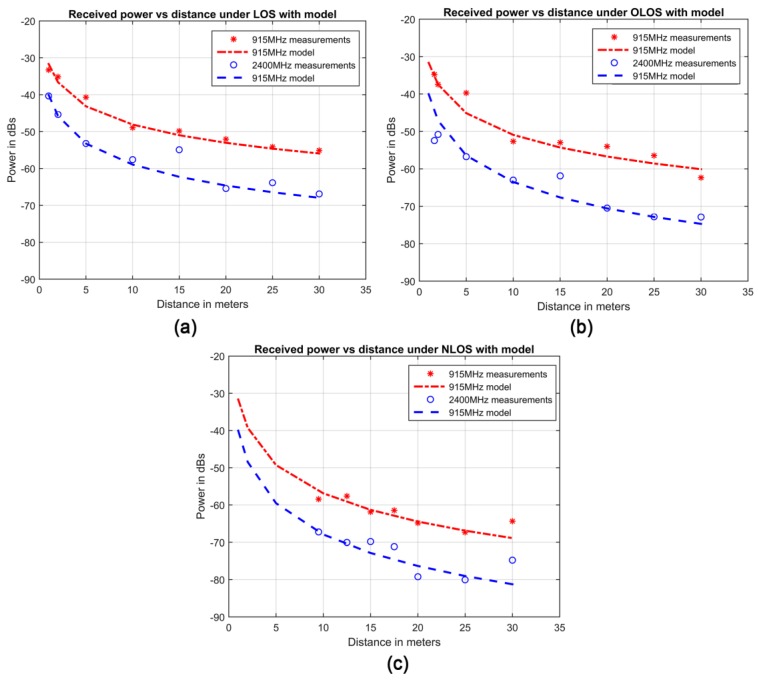
Acquired data and obtained model for (**a**) LOS, (**b**) OLOS, and (**c**) NLOS and both frequency bands.

Figure 4 shows the good fit of the LNSPL model to the data. The depicted markers represent the acquired data (i.e. the average values of Figure 3) whereas the continuous lines illustrate the fitted model (whose formulae for each scenario are detailed in Table 1).

Aside from the received power at a specific distance, the presence of electromagnetic interferences (EMI) has also been analyzed for both frequency bands. This is of paramount importance as EMI plays an important role in scenarios like substations where there are many potential sources of background noise (such as switchgears, transformers, etc.) [40,41]. A FSH3 spectrum analyzer (Rhode & Schwarz^®^, Munich, Germany) has been used to this end.

Although each medium/high voltage substation might be different, most of them share many common features such as the presence of big-sized metallic structures (transformers, switchgears, etc.) at both sides of clear corridors (passageways) with overhead metallic wires (the bus bars, overhead lines, etc.) all along them. Furthermore, the minimum distance between energized structures (such as transformers) and the passageways are defined by strict security regulations (such as the High Voltage Protection Action initiative of PSEG [42], the HV design considerations of the IEEE Houston Section [43], etc.). These two key aspects make many substations’ layout share, indeed, common features. And, although the specific disposition of certain elements may have an impact on propagation parameters, the rough propagation numbers in such substations will remain approximately the same.

### 3.3. Improvements on the TOSSIM Simulator

Traditionally, the TOSSIM simulator has been used to evaluate well-defined, homogeneous scenarios, i.e., ones where all nodes experience the same propagation phenomena. However, in complex and heterogeneous settings like SG’s substations, different visibility conditions and propagation phenomena may be experienced. These differences lead to variations in propagation losses and/or noise conditions that cannot be neglected by modern simulations. Another issue worth looking into it is the fact that TOSSIM has always been geared towards the evaluation of 2.4 GHz links; and hence, it lacks the necessary mechanisms to evaluate other WSN/IoT working frequencies.

Therefore, to accurately evaluate the network under study, we have extended the TOSSIM simulator to: (i) be able to embrace the heterogeneous nature of a SG and (ii) allow different working frequencies for deployed IoT devices.

The first task translates into modifying TOSSIM to permit the presence of different nodes working under various visibility conditions (e.g., the node pairs 0-4 of Figure 2a work under line-of-sight, whereas 0-3 may operate under NLOS conditions). By modifying TOSSIM’s architecture, we enabled each node pair to have its own combination of *n* (path-loss exponent) and σ (standard deviation). This makes the simulation environment more flexible. Furthermore, in order to simulate different frequencies, it is also required to generate distinct BPL values according to the working frequency under use. The second issue is addressed to provide the appropriate signal modulation scheme in accordance with the frequency band. The reason is obvious; 915 MHz transceivers usually employ different modulation schemes than 2400 MHz transceivers. Hence, when the 915 MHz band is under use, the frequency-shift keying (FSK) modulation scheme is utilized (the one typically exploited by transceivers operating in such a band), whereas when transmitting in the 2400 MHz band, the OQPSK (offset quadrature phase-shift keying) modulation scheme is used. Both, FSK and OQPSK, have been implemented into TOSSIM simulator. The full modifications carried out in the TOSSIM simulator, along with the automatization scripts are at the disposal of interested readers in [25].

## 4. 915 MHz/2400 MHz Bands’ Performance in SG-oriented IoTs

In this section, we present and discuss the performance results, in terms of the three key performance indicators, of the two scenarios evaluated. We start by listing the variables employed in the Scenario 1 (the one with 2.4 GHz co-existing devices) and the obtained results. Then, the noise conditions are altered (Scenario 2) to show a different condition on which an SG-oriented IoT may operate.

Both scenarios share some common parameters: the payload lengths assessed (6, 12, 18, and 24 bytes), the scaling factors (α) evaluated (0.5 to 1.5 in steps of 0.05), the transmission rate of each band (40 Kbps for the 915 MHz band and 250 Kbps for the 2400 MHz one, as defined by the 802.15.4 standard), and the packet generation rate (modeled via a random uniform distribution which generates a packet every 1 to 3 s). Data generation rate, payload length and α ranges are in line with the nature of SG-oriented IoT services and are chosen so as to effectively illustrate when it is more beneficial to work in one band or another. Furthermore, the above packet lengths reproduce the wide variety of services that SG-oriented IoT may offer: from very limited reports that may fit in payloads of six bytes, such as informing of a particular switchgear temperature, to more complex ones that may need (one or more) 24-byte packets, such as a complete report of a transformer status.

### 4.1. Scenario 1: Presence of 2400 MHz Co-Existing Devices

In this scenario, we consider an environment in which the 2400 MHz band is extensively employed by many other devices and hence, suffers moderate interference levels with an average noise floor of −87.4 dB. The 915 MHz band, on the other hand, as it is not usually shared with other technologies nowadays, remains relatively free from interference, presenting an average noise floor of −93.85 dB. These exact noise values have been obtained from the real SG under consideration using the Rhode & Schwarz^®^ FSH3 spectrum analyzer and are incorporated in the TOSSIM simulator.

**Figure 5:**
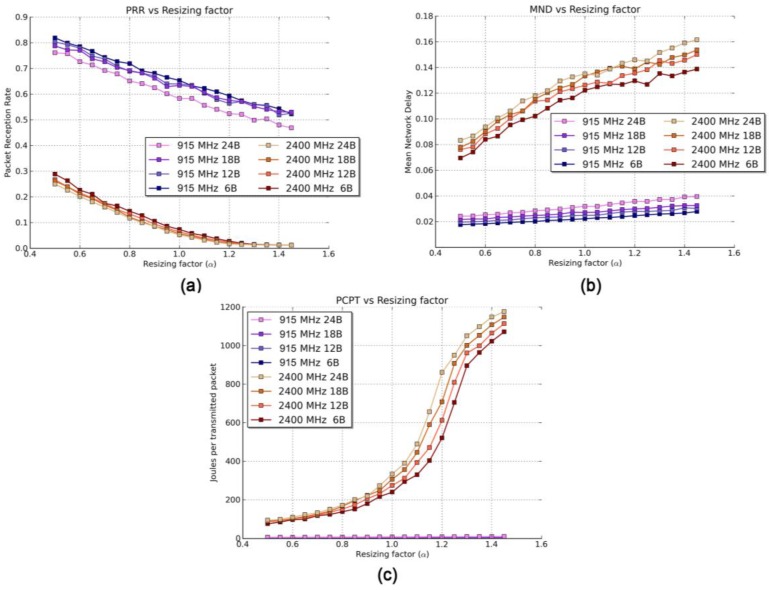
Results of the Scenario 1 for different packet sizes and resizing factors. (**a**) PRR, (**b**) MND, and (**c**) PCPT.

The results obtained are shown in Figure 5a for the packet reception rate (PRR), Figure 5b for the mean network delay (MND), and Figure 5c for power consumption per packet transmission (PCPT).

The first and most notable highlight of Figure 5a is the good behavior of the 915 MHz band (blue, upper lines) compared with the 2400 MHz band (red, lower lines) in terms of PRR. The reason for this is threefold: firstly, differences in LNSPL models (presented in Table 1) reveal the tendency of the 2400 MHz band to attenuate signals more than the 915 MHz band. Secondly, the 2400 MHz band experiences a higher noise floor level due to the presence of other co-existing devices. Finally, the OQPSK modulation (employed in the 2400 MHz band) tends to perform slightly worse in terms of PRR versus signal-to-noise ratio than the FSK modulation (used in the 915 MHz band) [44].

Both bands face the typical effects of the distance on the PRR. When α (and thus ${\bar{D}}_{in}$) increases, the received power decreases, leading to a larger number of packet losses and a decrease of the PRR. However, this KPI is not affected evenly in both bands, as the 2400 MHz band deteriorates slightly faster than the 915 MHz band due to higher path-losses. Regarding the packet size, larger packets produce smaller PRR values; given a certain bit error rate, an increment of the payload length always implies a smaller PRR. However, we can claim that packet size is not a crucial variable as it entails a PRR drop of less than 5%, on average, when increasing the payload length from 6 bytes to 24 bytes.

When the number of packet losses increases (and hence, the PRR decreases) a higher number of retransmissions is issued. This leads to an increment of the MND as this KPI takes into account the extra time taken by retransmissions (Figure 5b). Therefore, it should be noted that even though the transmission rate of the 2400 MHz band is higher, due to big differences in terms of PRR, the MND of the 2400 MHz band is, on average, four times larger than that obtained for the 915 MHz band. Under these circumstances, this phenomenon makes the 2400 MHz band less adequate for delay-sensitive applications. As with the PRR, an increment of the scaling factor (α) negatively affects the MND in both bands. Again, due to differences in path-losses, the 2400 MHz band is much more sensitive to α than the 915 MHz band. Considering the dependency of MND with α, the 2400 MHz band grows seven times faster than the 915 MHz band.

Figure 5c illustrates the PCPT. The very low value for the 2400 MHz band is, again, produced by the large volume of retransmissions, which greatly increases the power consumption. The 2400 MHz-band consumption is up to 120 times larger than the 915 MHz one (1187 joules versus 9.7 joules per packet transmission for a packet length of 24 B and α = 1.5). Therefore, the latter band should become the preferred choice for energy-constrained IoT deployments.

### 4.2. Scenario 2: Absence of 2400 MHz Co-Existing Devices

A capital factor in the performance of wireless networks is their coexistence with other devices operating in the same frequency band. This second scenario evaluates the situation in which an IoT is deployed in an environment that presents the same amount of interference in both bands (set to −93.85 dB).

**Figure 6:**
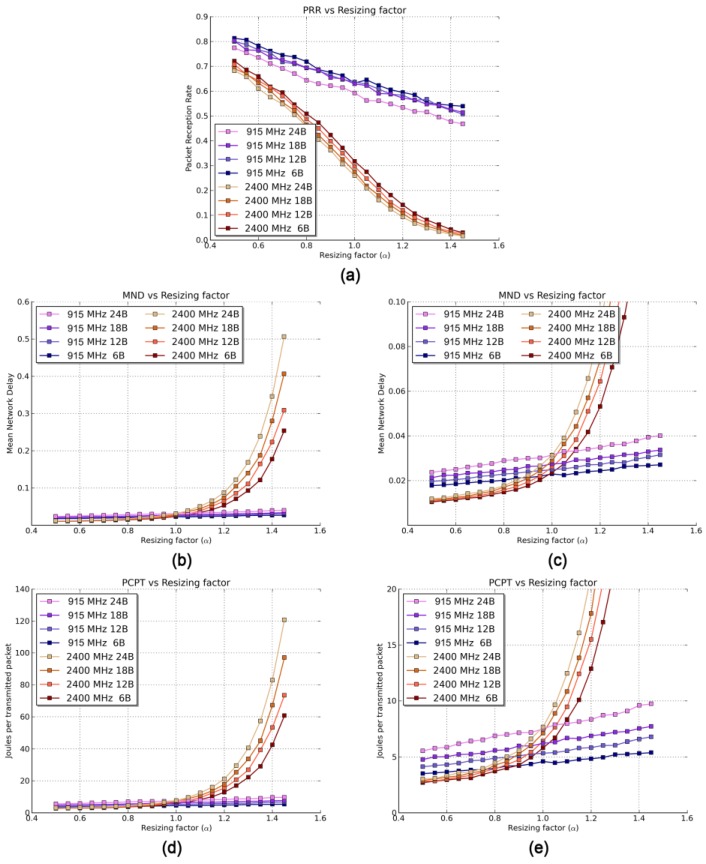
Results of Scenario 2 for different packet sizes and resizing factors. (**a**) PRR, (**b**) MND, (**c**) Zoom of MND, (**d**) PCPT, and (**e**) Zoom of PCPT.

This will generally be the case in which the SG is placed in a remote area—hence, the absence of other interfering 2400 MHz devices. The rest of the variables will remain unaltered. As in the previous scenario, the PRR is greater for the 915 MHz band than for the 2400 MHz one (Figure 6a). However, the sheer differences that appeared in Scenario 1 are no longer present when both bands are subject to the same noise conditions. Nevertheless, the PRR for the 2400 MHz band is still much more affected by the resizing factor due to, mainly, a larger path loss, decreasing 2.25 times faster with α than for the 915 MHz band.

Figure 6b shows a general outlook of the MND results, whereas Figure 6c depicts a close-up of the first α points. The results reveal a very interesting fact: in terms of MND, there are distances for which the 2400 MHz band is better suited than the 915 MHz one (and vice versa). When communicating devices are relatively far apart (e.g., greater than α = 0.996 or ${\bar{D}}_{in}$ = 7.7 m for a packet length of 6 B), the increment of path losses in the 2400 MHz band cannot be compensated by the higher transmission rate, thus resulting in larger MND values in this band. Conversely, when the resizing factor is small, the short number of packet losses does not play a major role in the MND, since the transmission rate is a more determining factor and presents a smaller value for the 915 MHz band. Therefore, when designing delay-sensitive networks, one should consider not only the transmission rate of the chosen band but also the ${\bar{D}}_{in}$ of the deployment, incorporating it as a key design parameter.

Since both the MND and the PCPT are affected by the number of retransmissions, they are strongly influenced by the packet losses, evaluated by the PRR. Therefore, the PCPT shows a similar tendency with α than the MND. Again, there are specific values of α for which SG-oriented IoT operating in the 2400 MHz band will benefit from a lower power consumption and vice versa. For example, for 6-byte long payloads, devices working in the 2400 MHz band will consume less energy if the network presents a small ${\bar{D}}_{in}$ (specifically, values of ${\bar{D}}_{in}$ smaller than 6.96 m or α = 0.9). This is explained by the behavior of the PRR along with the time that the transceiver requires to transmit a packet. When the PRR is relatively high for both bands, the much shorter transmitting times of the 2400 MHz band make the total power consumption smaller; power consumption strongly depends on the time the device is actively sending a packet. However, when the PRR drops abruptly (α > 0.9), these shorter times cannot compensate for the large number of losses.

Furthermore, since bigger packets take longer times, the 2400 MHz especially benefits from a faster transmission rate when transmitting larger payloads. Finally, as α grows, the power consumption in the 2400 MHz band greatly increases due to a corresponding decrease of the PRR. This increment leads to a PCPT up to 12 times higher for the 2400 MHz band than for the 915 MHz one for a 24-byte long payload.

## 5. Conclusions

A comprehensive study of how the working frequency band (915 MHz or 2400 MHz) affects IoT networks operating in SGs has been conducted. To do so, we have proposed a dual-band propagation model based on the well-established LNSPL model. Our model implementation has been geared towards IoT-based SG networks and with a special focus on radio propagation visibility conditions (LOS/OLOS/NLOS). Accordingly, we have extended the TOSSIM simulator to incorporate this new propagation model and developed a set of tools to efficiently extract data from a series of simulations.

By defining specific KPI (PRR, MND, and PCPT), we have quantified the performance of the IoT network under different scenarios. In general terms, we can unexpectedly affirm that in SG scenarios where the 2400 MHz band is used by other devices, the 915 MHz band is always the best option in terms of the three analyzed KPI, regardless of the packet length, and ${\bar{D}}_{in}$ (mean inter-node distance). However, when both bands are equally affected by a similar noise level, there are particular values of ${\bar{D}}_{in}$ for which each band is better suited: the 2400 MHz band is the preferred option for small size networks, whereas for large size networks the 915 MHz band is the best choice.

Although the exact values of when to use one or another band will depend on the specific environment and network layout, the models and figures presented here seek to raise awareness and set ground rules for when to consider the proper standardized ISM band in SG environments as the best option.

## Figures and Tables

**Table 1:** Obtained propagation model's parameters for both frequency bands and different visibility conditions.

Scenario	LNSPL Model
915 MHz – LOS	$PL{\left(d\right)}_{dB}=31.67+10\cdot 1.642\cdot \log_{10}\left(d\right)+{Χ}_{2.876}$
915 MHz – OLOS	$PL{\left(d\right)}_{dB}=31.67+10\cdot 1.924\cdot \log_{10}\left(d\right)+{Χ}_{3.131}$
915 MHz – NLOS	$PL{\left(d\right)}_{dB}=31.67+10\cdot 2.520\cdot \log_{10}\left(d\right)+{Χ}_{3.033}$
2400 MHz – LOS	$PL{\left(d\right)}_{dB}=40.05+10\cdot 1.888\cdot \log_{10}\left(d\right)+{Χ}_{4.775}$
2400 MHz – OLOS	$PL{\left(d\right)}_{dB}=40.05+10\cdot 2.346\cdot \log_{10}\left(d\right)+{Χ}_{4.582}$
2400 MHz – NLOS	$PL{\left(d\right)}_{dB}=40.05+10\cdot 2.788\cdot \log_{10}\left(d\right)+{Χ}_{3.962}$

## References

[1] Yun M., Yuxin B. Research on the architecture and key technology of Internet of Things (IoT) applied on smart grid. Proceedings of the International Conference on Advances in Energy Engineering.

[2] Blumsack S., Fernandez A. (2012). Ready or not, here comes the smart grid!. Energy.

[3] Martinez-Sandoval R., Garcia-Sanchez A.J., Garcia-Sanchez F., Garcia-Haro J., Flynn D. (2014). A comprehensive WSN-based approach to efficiently manage a smart grid. Sensors.

[4] Bellavista P., Cardone G., Corradi A., Foschini L. (2013). Convergence of MANET and WSN in IoT urban scenarios. IEEE Sens. J..

[5] Hossen M., Jang B.J. Extension of wireless sensor network by employing RoF-based 4G network. Proceedings of the 11th International Conference on Advanced Communication Technology.

[6] Eddabbah M., Moussaoui M., Laaziz Y. A flexible 3G WebService based gateway for wireless sensor networks in support of remote patient monitoring systems. Proceedings of the 2014 Mediterranean Microwave Symposium (MMS2014).

[7] Akyildiz I.F., Lee W.-Y., Vuran M.C., Mohanty S. (2006). NeXt generation/dynamic spectrum access/cognitive radio wireless networks: A survey. Comput. Netw..

[8] Akan O.B., Karli O.B., Ergul O. (2009). Cognitive radio sensor networks. IEEE Netw..

[9] Yang Z., Shi Z., Jin C. (2016). SACRB-MAC: A High-Capacity MAC Protocol for Cognitive Radio Sensor Networks in Smart Grid. Sensors.

[10] Gharghan S.K., Nordin R., Ismail M. (2014). Energy-Efficient ZigBee-Based Wireless Sensor Network for Track Bicycle Performance Monitoring. Sensors.

[11] Lopez-Iturri P., Aguirre E., Azpilicueta L., Astrain J.J., Villadangos J., Falcone F. (2016). Implementation and Analysis of ISM 2.4 GHz Wireless Sensor Network Systems in Judo Training Venues. Sensors.

[12] Tuwanut P., Kraijak S. A survey on IoT architectures, protocols, applications, security, privacy, real-world implementation and future trends. Proceedings of the 16th International Conference on Communication Technology.

[13] Gubbi J., Buyya R., Marusic S., Palaniswami M. (2013). Internet of Things (IoT): A vision, architectural elements, and future directions. Future Gener. Comput. Syst..

[14] Parikh P.P., Kanabar M.G., Sidhu T.S. Opportunities and challenges of wireless communication technologies for smart grid applications. Proceedings of the 2010 IEEE Power and Energy Society General Meeting.

[15] Ma R., Chen H.H., Huang Y.R., Meng W. (2013). Smart grid communication: Its challenges and opportunities. IEEE Trans. Smart Grid.

[16] Fadel E., Gungor V.C., Nassef L., Akkari N., Maik M.G.A., Almasri S., Akyildiz I.F. (2015). A survey on wireless sensor networks for smart grid. Comput. Commun..

[17] Kilic N., Gungor V.C. (2013). Analysis of low power wireless links in smart grid environments. Comput. Netw..

[18] Palattella M.R., Accettura N., Grieco L.A., Boggia G., Dohler M., Engel T. (2013). On Optimal Scheduling in Duty-Cycled Industrial IoT Applications Using IEEE802.15.4e TSCH. IEEE Sens. J..

[19] Molisch A.F., Balakrishnan K., Chong C., Emami S., Fort A., Karedal J., Kunisch J., Schantz H., Schuster U., Siwiak K. IEEE 802.15.4a Channel Model. http://www.ieee802.org/15/pub/2004/15-04-0662-00-004a-channel-model-final-report-r1.pdf.

[20] Subramanian A.P., Gupta H., Das S.R., Cao J. (2008). Minimum Interference Channel Assignment in Multiradio Wireless Mesh Networks. IEEE Trans. Mobile Comput..

[21] Wu D., Bao L., Liu C.H. (2013). Scalable Channel Allocation and Access Scheduling for Wireless Internet-of-Things. IEEE Sens. J..

[22] Kim H. Low power routing and channel allocation method of wireless video sensor networks for Internet of Things (IoT). Proceedings of the IEEE World Forum on Internet of Things (WF-IoT).

[23] Güzelgöz S., Arslan H., Islam A., Domijan A. (2011). A Review of Wireless and PLC Propagation Channel Characteristics for Smart Grid Environments. J. Electr. Comput. Eng..

[24] Levis P., Lee N., Welsh M., Culler D. TOSSIM: Accurate and Scalable Simulation of Entire TinyOS Applications. Proceedings of the 1st International Conference on Embedded Networked Sensor Systems.

[25] Sandoval R.M., Garcia-Sanchez A.-J., Garcia-Sanchez F., Garcia-Haro J. Propagation Model and Tools. http://labit501.upct.es/~rmartinez/915vs2400/.

[26] Instruments N. National Instruments—Coherence Bandwidth. http://www.ni.com/white-paper/14910/en/.

[27] Karnouskos S. The cooperative internet of things enabled smart grid. Proceedings of the 14th IEEE international symposium on consumer electronics (ISCE2010).

[28] Bonomi F., Milito R., Zhu J., Addepalli S. Fog Computing and Its Role in the Internet of Things. Proceedings of the First Edition of the MCC Workshop on Mobile Cloud Computing.

[29] Kusy B., Richter C., Hu W., Afanasyev M., Jurdak R., Brunig M., Abbott D., Huynh C., Ostry D. Radio diversity for reliable communication in WSNs. Proceedings of the 10th ACM/IEEE International Conference on Information Processing in Sensor Networks.

[30] Hrovat A., Javornik T. Radio channel models for wireless sensor networks in smart city applications. Proceedings of the 2013 International Conference on Electronics, Signal Processing and Communication Systems.

[31] Ferrer Coll J., Dolz Martin de Ojeda J., Stenumgaard P., Marzal Romeu S., Chilo J. Industrial indoor environment characterization—Propagation models. Proceedings of the 10th International Symposium on Electromagnetic Compatibility.

[32] Rao T.R., Balachander D., Nishesh T., Prasad M. (2014). Near ground path gain measurements at 433/868/915/2400 MHz in indoor corridor for wireless sensor networks. Telecommun. Syst..

[33] Tanenbaum A.S. (2003). Computer Networks. Computer Networks.

[34] Tanghe E., Joseph W., Bruyne J.D., Verloock L., Martens L. (2010). The industrial indoor channel: Statistical analysis of the power delay profile. AEU Int. J. Electron. Commun..

[35] Rappaport T.S. (1989). Characterization of UHF multipath radio channels in factory buildings. IEEE Trans. Antennas Propag..

[36] Rappaport T.S., MacCartney G.R., Samimi M.K., Sun S. (2015). Wideband Millimeter-Wave Propagation Measurements and Channel Models for Future Wireless Communication System Design. IEEE Trans. Commun..

[37] Latsoudas G., Sidiropoulos N.D. (2007). A Fast and Effective Multidimensional Scaling Approach for Node Localization in Wireless Sensor Networks. IEEE Trans. Signal Process..

[38] Garg R., Varna A.L., Wu M. (2012). An Efficient Gradient Descent Approach to Secure Localization in Resource Constrained Wireless Sensor Networks. IEEE Trans. Inf. Forensics Secur..

[39] Yao L., Sheng Q.Z., Ngu A.H.H., Ashman H., Li X. Exploring Recommendations in Internet of Things. Proceedings of the 37th International ACM SIGIR Conference on Research & Development in Information Retrieval.

[40] Shapoury A., Kezunovic M. Noise Profile of Wireless Channels in High Voltage Substations. Proceedings of the 2007 IEEE Power Engineering Society General Meeting.

[41] Sallabi F.M., Gaouda A.M., El-Hag A.H., Salama M.M.A. (2014). Evaluation of ZigBee Wireless Sensor Networks Under High Power Disturbances. IEEE Trans. Power Deliv..

[42] PSEG–High Voltage Protection Action. https://www.pseg.com/business/local_government/safety/pdf/HighVoltageProximityActGov.pdf.

[43] IEEE Houston Section—High Voltage Substation Application Design. http://sites.ieee.org/houston/files/2016/04/2012-10-02-HV-Substation-Application-Design-Oct-2-3.pdf.

[44] Vuran M.C., Akyildiz I.F. (2009). Error Control in Wireless Sensor Networks: A Cross Layer Analysis. IEEE/ACM Trans. Netw..

